# WHAT TYPES OF SUPPORT BURDENED DEMENTIA CAREGIVERS LOOK FOR?

**DOI:** 10.1093/geroni/igae098.3328

**Published:** 2024-12-31

**Authors:** Yeji Hwang, Hannah Cho

**Affiliations:** Seoul National University, Seoul, Republic of Korea; University of Pennsylvania, Philadelphia, Pennsylvania, United States

## Abstract

As dementia caregivers provide excessive amount of care daily to persons living with dementia, many caregivers feel physical or emotional burden. However, lack of information is available on what kind of support that caregivers seek for when they feel physical or emotional burden. Therefore, this study aimed to investigate support that burdened dementia caregivers look for. We conducted a secondary data analysis of the National Study of Caregiving (NSOC) IV, focusing on caregivers of persons living with possible or probable dementia (n=518). Descriptive data analyses, chi square tests, and logistic regression analyses were performed for data analysis. In this study, when caregivers felt emotional burden (42%), they were more likely to use service that take care of the care recipient so that they could take some time away from caregiving (OR= 3.160, p=0.001) or find paid helper to do household chores or personal care (OR= 2.259, p=0.004). Similarly, when caregivers felt physical burden (23.98%), they were more likely to use service that take care of the care recipient so that they could take some time away from caregiving (OR= 3.050, p=0.004) or find paid helper to do household chores or personal care (OR= 2.651, p=0.006). Other support such as support group or training for caregivers did not show any significance. This study found dementia caregivers seek support services when they feel emotional or physical burden. Policy makers should consider these services readily accessible to dementia caregivers.

